# Characterization of Co-Stimulatory Ligand CD80/86 and Its Effect as a Molecular Adjuvant on DNA Vaccine Against *Vibrio anguillarum* in Flounder (*Paralichthys olivaceus*)

**DOI:** 10.3389/fimmu.2022.881753

**Published:** 2022-05-10

**Authors:** Wenjing Liu, Jing Xing, Xiaoqian Tang, Xiuzhen Sheng, Heng Chi, Wenbin Zhan

**Affiliations:** ^1^ Laboratory of Pathology and Immunology of Aquatic Animals, Key Laboratory of Mariculture, Ministry of Education (KLMME), Ocean University of China, Qingdao, China; ^2^ Laboratory for Marine Fisheries Science and Food Production Processes, Qingdao National Laboratory for Marine Science and Technology, Qingdao, China

**Keywords:** CD80/86, lymphocytes, adaptive immune, adjuvants, flounder

## Abstract

The CD80/86 molecule is one of the important co-stimulatory ligands and involves antigen-specific immune responses by ligating with CD28 and then delivering the required second signal to T-cell activation. In this study, a CD80/86 homolog was identified, and its expression characteristics were studied in flounder (*Paralichthys olivaceus*). The open reading frame (ORF) of *CD80/86* is 906 bp, encoding 301 aa, and the extracellular amino acid sequence encoded two IgV- and IgC-like structural domains; *fCD80/86* is highly expressed in head kidney, peripheral blood leukocytes (PBLs), and spleen, and has relatively high expression in muscle. Antibodies specific for CD80/86 were produced, and CD80/86 was colocalized with MHCII+, CD40+, and CD83+ leukocytes but not with IgM+, CD3+, or CD4+ lymphocytes. The cloned CD80/86 in flounder shares conserved structural features with its mammalian counterparts and is mainly distributed on antigen-presenting cells. Based on these data, CD80/86 as an adjuvant to enhance the immune response of DNA vaccine was investigated. A bicistronic DNA vaccine expressing both CD80/86 and the outer membrane protein (OmpK) of *Vibrio anguillarum* (p-OmpK-CD80/86) was successfully constructed. After immunization, p-OmpK-CD80/86 could induce the upregulation of the proportion of IgM+ and CD4+ cells in flounder, compared to the p-OmpK- or p-CD80/86-immunized group; *CD28* genes were significantly induced in the p-CD80/86 and p-OmpK-CD80/86 groups. Compared to the p-OmpK group, the higher expression of *CD83*, *MHCI*, *CD4*, *CD8*, and *IL-2* was detected at the injection site. The relative percent survival (RPS) produced by p-OmpK-CD80/86 is 66.11% following the *V. anguillarum* challenge, while the RPS of p-OmpK or p-CD80/86 is 46.30% and 5.56%, respectively. The results revealed that CD80/86 is mainly found in antigen-presenting cells, and could help elicit humoral immune responses in teleost through the CD80/86-CD28 signaling pathway involving CD4+ lymphocytes.

## Introduction

T-cell immunity is regulated by antigen-specific recognition signals delivered by the T-cell receptor (TCR) binding to its specific MHC-peptide ligand and co-stimulatory molecules, especially B7/CD28 molecule-mediated co-stimulatory signaling (1, 2). More B7 family members have been identified in vertebrates (3–5); among them, the interaction between CD80/86 and its ligands is widely recognized as a major T-cell co-stimulatory pathway to regulate the immune response of T cells (2, 6). Both B7-1 and B7-2 are type-1 transmembrane glycoproteins that contain two extracellular Ig-like domains (7, 8). B7-1 is present as a dimer and is mainly found on activated B cells, activated T cells, and macrophages, while CD86 exists as a monomer and is thought to be more widely expressed at higher levels than CD80, which is constitutively expressed in dendritic cells (DCs), Langerhans cells, peripheral blood DCs, memory B cells, and germinal center B cells (9). Even though they have structural similarities and are both coded on human chromosome 3q21, only 25% of the amino acids’ identity is shared between them (10).

The ligation between CD80/CD86 and CD28 delivers co-stimulatory signals that activate initial T cells and promote T-cell proliferation, interleukin 2 (IL-2) production, prevention of anergy, and induction of the anti-apoptotic factor Bcl-xL (11, 12). In contrast, CD152 (CTLA-4), another ligand of CD80/86, binds to CD80/CD86 with a higher affinity to mediate co-inhibitory signals to regulate T-cell immunity (13, 14). CD86 is a more important co-stimulatory ligand than CD80 for T-cell activation as demonstrated by knockout mouse models (15), and CD80 is more clearly involved in the immune regulation of T cells by interacting with CTLA-4 (16). In contrast, different concepts point out that CD86 involves both proliferation mediated by infected cells and that mediated by cells pulsed with parasite-soluble Ags, and CD80 only participates in the T-cell proliferation in the *T. gondii*-mediated proliferation of T-cell assay (17, 18). Although CD80 and CD86 have similar structures, their distribution characteristics, unique cell surface aggregation status, and different regulation of the immune response by CD80 and CD86 show the differences between them.

Intriguingly, only one B7 transcript, namely, CD80/86, has been identified in most bony teleost including yellow catfish (*Pelteobagrus fulvidraco*), Nile tilapia (*Oreochromis niloticus*), grouper (*Epinephelus coioides*), and zebrafish (*Danio rerio*) (19–22). Notably, more forms of CD80/86 have been identified in rainbow trout (*Oncorhynchus mykiss*), and both rtCD80/86A and rtCD80/86B contain a membrane-bound form and two soluble forms, respectively (19, 20). The distribution of CD80/86 molecules varies considerably among different tissues in fish, mainly detected in lymphoid tissues. Moreover, stimulation by pathogenic bacteria and parasites was able to induce significant changes in CD80/86, suggesting that CD80/86 plays an important role in fish immunity. Moreover, CD80/86 has been proven to be located on B lymphocytes in the zebrafish model and be essential for B-cell-initiated adaptive immunity (22). From the evolution, distribution characteristics, and role of CD80/86 in immune regulation, CD80/86 may play an immune role similar to mammalian CD86 than to CD80 in fish.

DNA vaccines induce immunoprotected responses by enabling the expression of antigenic proteins *in vivo* from plasmids encoding antigens. Diverse administration methods of DNA vaccines have been developed, and the most common method is still intramuscular injection (23–25). However, unlike protein antigens, for a DNA vaccine to work effectively, it must enter the cytoplasm at the injection site to induce antigen expression *in vivo*. When a DNA plasmid is injected into a muscle, the DNA plasmid enters the cell and translocates to the nucleus, and the antigen is expressed by the transfected cell. In most cases, myocytes and antigen-presenting cells (APCs), such as DCs or macrophages, are involved in capturing the plasmid DNA. Subsequently, the antigen protein is degraded and delivered by MHC-I in immune cells. In addition, the expressed antigen can be secreted from the cells by active protein secretion or released due to the apoptosis of transfected cells. Secreted antigen proteins are taken up, degraded, and delivered by APCs on MHC-I and MHC-II molecules. Finally, APCs activate naive B cells and CD4+ and CD8+ T cells, contributing to the activation of cellular and humoral immune responses. A better understanding of the mechanism of action of DNA vaccines also allows us to better exploit the intrinsic host response to DNA to enhance the immunogenicity of vaccines (26, 27).

DNA vaccines against infectious hematopoietic necrosis virus (IHNV), viral haemorrhagic septicemia virus (VHSV), and infectious pancreatic necrosis virus (IPNV) have been applied to reduce the occurrence of viral diseases in trout farming (28, 29). Although DNA vaccines are safe, easy to construct, and capable of inducing a specific immune response, especially T-cell responses, low delivery efficiency and weak immunogenicity are still key factors hindering the application of DNA vaccines. To improve the immunogenicity of DNA vaccines, adjuvants have been co-administered *in vivo* with DNA vaccines (30, 31). Except for the traditional adjuvants such as aluminum salts, polysaccharides, CpG oligonucleotides, chemical molecules, and liposomes, molecular adjuvants like cytokines, chemokines, or co-stimulatory molecules also have an excellent adjuvant effect on DNA vaccine (30, 32). The chemokines CCL3, CCL4, CCL19, and CCL21 have been proven to enhance the immunoprotection of the VAA DNA vaccine against *V. anguillarum* (33). The fish co-injected glycoprotein with IL-2, IL-8, IL-15, or IL-17 plasmids enhanced the protection of trout against IHNV, and the interleukin exhibited a stronger induction of lymphocyte proliferation (34). However, the application of co-stimulatory molecules as adjuvants for DNA vaccines in fish has not been studied.

Therefore, in the present study, CD80/86 homologs have been cloned in flounder (*Paralichthys olivaceus*), and the distribution characteristics of CD80/86 in different leukocyte subpopulations were investigated by the prepared anti-CD80/86-specific antibodies. In addition, a bicistronic DNA vaccine expressing both CD80/86 and OmpK of *Vibrio anguillarum* (p-OmpK-CD80/86) was constructed to evaluate co-stimulatory molecules as DNA vaccine adjuvants in teleost.

## Materials and Methods

### Experimental Animal, Antibodies, and Bacteria

Flounder (*P. olivaceus*) of both sexes, with an average weight of 50 ± 5 g and with a length of 12 ± 1.3 cm, were obtained from a farm in Qingdao, China. During the entire animal trial, flounder were acclimated in aquarium tanks using a recirculating seawater system at 20 ± 2°C and fed with commercial dry food pellets three times a day. All the experimental treatment were performed under natural 12-h light and dark cycle. After acclimation to the laboratory setting for 7 days, the healthy fish were used for subsequent experiments. New Zealand white rabbits (∼1 kg) and Balb/C(~30 g)mice were purchased from the Qingdao Animal Experimental Center (Shandong, China) and then used for antibody production. All animal work in this paper was conducted according to relevant national and international guidelines. Fish were anesthetized with tricaine methanesulfonate (MS222, Sigma, USA) before immunization, tissue collection, and challenge.

Polyclonal antibodies against flounder CD83, CD40, and MHCII, and monoclonal antibodies against flounder IgM and CD4 were previously produced in our laboratory (35–38), and all antibodies were diluted 1:1,000. The rabbit against OmpK antibodies were produced by Li et al. (39). The secondary antibodies included Dylight 488 (Abbkine; diluted 1:1,000) Rabbit or mouse IgG, Dylight 649 (Abbkine; diluted 1:1,000) Rabbit or mouse IgG, alkaline phosphatase (AP)-conjugated goat anti-rabbit IgG (H + L) (Abbkine; diluted 1:5,000), and HRP-conjugated goat anti-mouse IgG (Abbkine; diluted 1:5,000).

*V. anguillarum* (ATCC 43305/MH67) was isolated from diseased flounder (*P. olivaceus*) stored in the Laboratory of Pathology and Immunology of Aquatic Animals, Ocean University of China. The bacteria were grown in marine broth 2216E (5 g/L Tryptone, 1 g/L yeast extract, and 0.1 g/L C_6_H_5_Fe·5H_2_O, pH 7.6) at 28°C to OD_600_ = 0.2, and centrifuged at 2,000 *g* for 5 min to harvest the bacteria. Bacteria adjusted to different concentrations with PBS were used in the immunization experiment.

### Cloning of CD80/86

The CD80/86 sequence of *P. fulvidraco*, rainbow trout, *O. niloticus*, or zebrafish was used to search the flounder transcripts database published on National Center for Biotechnology Information (NCBI; http://www.ncbi.nlm.nih.gov) through BLASTn or BLASTp. Meanwhile, the published amino acid sequence of CD80/86 in teleost was compared to obtain the conserved sequence, and then the primers were designed to amplify the CD80/86 gene by using the head kidney cDNA of flounder. Based on the partial sequence searched, specific primers were designed by PrimerPremier 5.0 (Listed in **Table 1**) to extend 3’ and 5’ untranslated region (UTR) using cDNA from the spleen by rapid amplification of cDNA ends (RACE) method.

**Table 1:** Sequences of primers used for gene cloning.

Primer name	Sequence (5’-3’)	Application
***CD80/86***	**F:** CCTACAGCAAACCCACTGCGACA	Gene cloning
	**R:** CCACCTTGATCCCTGGGTAC	
***CD80/86-3’***	TGTCGCAGTGGGTTTGCTGTAG	3’ RACE
***CD80/86-5’***	CCTACAGCAAACCCACTGCGACA	5’ RACE
***CD80/86-E***	**F:** CCCAAGCTTAGTGCTATCACCATTCAACTC (*BamHI*)	Prokaryotic expression
	**R:** CGCGGATCCTTTGAGAGGAGTGTAAGAGTTAG (*HindIII*)	
***qCD80/86***	**F:** CAAGGTGGAGTGGATAAT	qPCR
	**R:** AGAACAAGTCGGAGGTAA	
***β-actin***	**F:** GAGGGAAATCGTGCGTGACAT	qPCR
	**R:** ATTGCCGATGGTGATGACCTG	

F, forward; R, reverse; RACE, rapid amplification of cDNA ends.

### Sequence Analysis

The full-length CD80/86 cDNA was assembled by DNAMAN. The potential open reading frame (ORF) was analyzed with the Finder program (https://www.ncbi.nlm.nih.gov/orffinder/). The signal peptide and the TM domain of the deduced protein sequences were predicted with the programs SignalP (http://www.cbs.dtu.dk/services/SignalP/) and TMHMM (http://www.cbs.dtu.dk/services/TMHMM/), respectively. Multiple sequence alignment was performed with the ClustalX program. Phylogenic trees were constructed using the neighbor-joining method with MEGA software (Version 6.0) and were bootstrapped 1,000 times. N-linked glycosylation sites were predicted with the NetGlycate Server (http://www.cbs.dtu.dk/services/). Secondary and 3D structures were analyzed using SMART, SWISS-MODEL, and I-TASSER (https://zhanglab.ccmb.med.umich.edu/I-TASSER/).

### Expression of *CD80/86* Gene in Flounder

The RNA was extracted from seven tissues, namely, the intestine, liver, peripheral blood leukocytes (PBLs), muscle, head kidney, spleen, and gills using Trizol Reagent (Invitrogen, USA). The first-strand synthesis used the Primerscript™ First Stand cDNA Synthesis kit (Takara, China). RT-qPCR was performed using the LightCycler^®^ 480 II Real-Time System (Roche, Switzerland) with ChamQ Universal SYBR qPCR Master Mix (Vazyme, China). The primers and housekeeping genes are listed in **Table 1**. Briefly, the cDNA was adjusted to 400 ng/ml. Each reaction consists of 10 μl of 2×ChamQ Universal SYBR qPCR Master Mix (Vazyme), 0.4 μl of forward and 0.4 μl of reverse primers (10 μM), 2 μl of cDNA, and 7.2 μl of ddH_2_O. The thermal cycling profile consisted of an initial denaturation at 95°C for 30 s, followed by 40 cycles of denaturation at 95°C for 10 s, and extension at 60°C for 30 s. An additional temperature ramping step was utilized to produce melting curves of the reaction from 60°C to 95°C. The *β-actin* gene was used as an internal control. The 2^−ΔΔCT^ method was used to analyze the expression level of the *CD80/86* gene.

### Preparation of CD80/86 Antibodies

The sequence encoding the extracellular domain except the signal peptide was amplified by polymerase chain reaction (PCR) and then embedded into pET-28a Vector (TaKaRa, Japan). After affinity purification of recombinant CD80/86 protein with a 6-histidine tag at the N-terminal end using His TrapTM HP Ni-Agarose (GE Healthcare China, Beijing, China), a total of 2.8 mg of recombinant protein was used to immunize New Zealand white rabbits (∼1 kg) and Balb/C mice (~30 g) (40). The specific procedure was followed as previously described. The antiserum was collected on the fifth day of the final immunization and purified by a protein A/protein G agarose column. The Abs titers and specificity were determined by ELISA, Western blot (WB), or mass spectrometry.

Western blotting was conducted as previously described (40). After SDS-PAGE, rCD80/86 and whole-cell protein extracts from PBLs were electrophoretically transferred onto a PVDF membrane (Merck Millipore). The membrane was blocked with 5% bovine serum albumin (BSA) in PBS for 1 h at 37°C, incubated with the rabbit anti-CD80/86 polyclonal antibodies or the unimmunized serum for 1 h at 37°C, and washed thrice with PBS-T, and then primary antibody was detected with AP-conjugated goat anti-rabbit IgG (H + L) or HRP-conjugated goat anti-mouse IgG (H + L) for 1 h at 37°C. After washing three times with PBST, the stained bands were captured by the Fusion FX Spectra (Vilber, France).

### Subcellular Localization Analysis

The leukocytes were isolated from peripheral blood by 34% (1.050 g/ml) to 51% (1.072 g/ml) v/v discontinuous Percoll density gradient. After being adjusted to 1 × 10^6^ cells/ml, 50 μl of cells was adhered to the slides for 2 h and then fixed with 4% paraformaldehyde for 20 min. The cells were blocked with 5% BSA at 37°C for 1 h. The CD80/86-specific antibodies mixed with mouse polyclonal antibodies MHCII (diluted 1:500), CD40, CD83, and monoclonal antibodies against flounder IgM, CD4-1, and CD4-2 were used as primary Abs at 37°C for 1.5 h. After three washes with PBS-T, the primary Abs were detected by Dylight 488 (Abbkine) rabbit or mouse IgG and Dylight 649 rabbit or mouse IgG. The nucleus was stained with 4,6-diamidino-2-phenylindole (DAPI) at room temperature for 20 min. All the Abs were diluted 1:1,000 and unimmunized serum was used as negative controls. Fluorescence images of the samples were obtained using a fluorescence microscope (Olympus DP70, Japan).

### Construction of Recombinant Eukaryotic Plasmids

The eukaryotic expression plasmid pBudCE4.1 (Invitrogen, Carlsbad, CA, USA) contains two promoters, human cytomegalovirus (CMV) and human elongation factor 1α-subunit (EF-1α), which were used as a vector to express exogenous genes CD80/86 and OmpK. The CD80/86 from flounder was ligated into the *Hind*III site located downstream of CMV, and OmpK from *V. anguillarum* was ligated into the *Kpn*I site located downstream of EF-1α (list in **Table 2**). The recombinant plasmids of pBudCE4.1, p-OmpK, p-CD80/86, and p-OmpK-CD80/86 were extracted using an EndoFree plasmid Kit (Tiangen, Beijing, China). The concentration of recombinant plasmid was adjusted to 200 μg/ml.

**Table 2:** Primer sequences for plasmid construction.

Primer	Primer sequence (5’-3’)	Accession No.
***CD80/86-F***	CGACTCACTATAGGGAGACCCAAGCTTATGGCATCCAGTTCCACCATACG	MT019837
***CD80/86-R***	GATGTCGACCTGCAGGAATGCTCAAGGTGCGTTAAAGTCATCTAC	
***OmpK-F***	TGGTCGCGGCCGCTTCGAAGGTACCATGCGTAAATCACTTCTAGCTC	FJ705222.1
***OmpK-R***	CCAGATCTCTCGAGTCCACTGTGCTGAACTTGTAAGTCACAGCTAGGTAG	
***CMV-F***	CGCAAATGGGCGGTAGeGCGTG	
***CMV-R***	GTGGTTTGTCCAAACTCATC	
***EF-1α-F***	TCAAGCCTCAGACAGTGGTTC	
***EF-1α-R***	TAGAAGGCACAGTCGAGG	

### Vaccination and Sampling

The healthy flounder were randomly divided into five groups (150 fish/group) and injected intramuscularly with 100 μl of pBudCE4.1, p-OmpK, p-CD80/86, and p-OmpK-CD80/86 recombinant plasmids, respectively. The equal volume of PBS was injected as a control treatment. To monitor changes in lymphocytes, the proportion of CD4 and IgM+ cells at different time points was examined by flow cytometry. The expression of recombinant plasmids in muscle was verified by indirect immunofluorescence. Variation of immune-related genes at the injection site was detected by qPCR.

### Immunofluorescence Staining

Three fish were selected from each group on the 7th day after immunization, and muscle tissue (~0.5 cm^3^) was taken from the injection site. The tissues were fixed with 4% paraformaldehyde at room temperature for 2–10 min and then dehydrated with 30% sucrose solution. The tissues were infiltrated using an OCT mixture (optimal cutting temperature compound, SAKURA) and frozen using liquid nitrogen. Tissues were cut into ~7-μm-thick sections using a Leica CM 1900 slicer (Leica, German). After being blocked with 5% BSA, the sections were incubated with rabbit anti-CD80/86 or anti-OmpK, respectively. Then, the frozen sections were incubated with Dylight 649 rabbit IgG antibodies and DAPI in turn. Unimmunized rabbit serum was used as the negative control.

### Flow Cytometry

The lymphocytes in peripheral blood were isolated by discontinuous Percoll gradient density (1.020–1.070 g/ml) on the 1st, 7th, and 14th day, and at weeks 3, 5, and 7. The lymphocytes were adjusted to 1 × 10^6^ cells/ml and incubated with anti-CD4 monoclonal antibodies (mixed with CD4-1 and CD4-2, diluted 1:1,000) and anti-IgM monoclonal antibodies (diluted 1:1,000). Then, the cells were stained with Dylight 488 (Abbkine) mouse IgG antibody at 37°C for 1 h. Flow cytometry was performed using FACSCalibur (BD Biosciences) flow cytometers and analyzed by FlowJo 10.7 (TreeStar).

### Variation of Immune-Related Genes at the Inoculation Site

The muscle of the injection site was sampled 72 h and 168 h after vaccination. The RNA was adjusted to 1,000 ng/μl and reversed to cDNA. The expression profiles of immune-related genes including CD4-1, CD8, CD28, TNF-α, IFN-γ, IL-2, CD83, MHCI, and MHCII were investigated. The qPCR primers are listed in **Table 3** and the amplification fragment size of the primers was between 150 and 250 bp and the amplification efficiency of the primers was within 90%–110%.

**Table 3:** Sequences of primers used for RT-PCR.

Gene’s name	Primer sequences (5’-3’)	GenBank accession no.
***CD4-1***	**F:** CAACTTGCCTGGCTCAATCACT	AB643634
	**R:** ACCGACTGGGTTCAAACTCACC	
***CD28***	**F:** TTCCAACGTCTCATGCACTGG	MT019836.1
	**R:** TTTTTGCTGTTTGCGCTCCAC	
***CD8β***	**F:** GATGACACTCAAACCTCCAGTCAA	AB643633
	**R:** GCCATCCTGTGCAAAATTCTTC	
***IL-2***	**F:** TAGAGGATGCCAGTATCGGTT	KY307833.1
	**R:** TACATTCTGCGGAGGTCGTTG	
***TNF-α***	**F:** TCCTGGCGTTTTCTTGGT	AB040448.1
	**R:** TGGCTCTGCTGCTGATTT	
***IFN-γ***	**F:** TGGTCTGTCTGTCCCTGTG	AB435093.1
	**R:** GCTTCCCGTTGAATCTGT	
***CD83***	**F:** CCCAACGGCACGACGACATAC	KR998303.1
	**R:** CCCAAAGGTGCTGCCAGGTGA	
***MHCIα***	**F:** AGACCACAGGCTGTTATCACCA	AB126921
	**R:** TCTTCCCATGCTCCACGAA	
***MHCIIα***	**F:** ACAGGGACGGAACTTATCAACG	AY99753
	**R:** TCATCGGACTGGAGGGAGG	
***β-actin***	**F:** GAGGGAAATCGTTCGTGACAT	AF135499.1
	**R:** ATTGCCGATGGTGATGACCTG	

### Challenge

Sixty flounder were randomly selected from the five immunized groups at week 7. The fish were intraperitoneally injected with 1.0 × 10^6^ CFU/fish (10×LD50) *V. anguillarum* after the last sampling. Mortalities were recorded within 30 days after the challenge. The relative percent survival (RPS) was calculated using the following formula: RPS = {1 − (% mortality in immunized fish/% mortality in control fish)} × 100%. The log rank method was used to detect the significant difference in survival rate between the groups.

### Data Analysis

Statistical analyses were performed using Statistical Product and Service Solution (SPSS) 20.0 software (IBM, Armonk, NY, USA) with one-way analysis of variance (ANOVA) followed by *t*-test or Duncan’s multiple range test. All data are expressed as the mean ± standard deviation (SD). Statistical parameters and details of experiments are provided in the figure legends, and exact *p*-values are shown in the figures. GraphPad Prism software version 5.01 (GraphPad 265 Software, San Diego, CA, USA) was used for plotting graphs.

## Results

### Characterization of CD80/86 in Flounder

**Figure 1:**
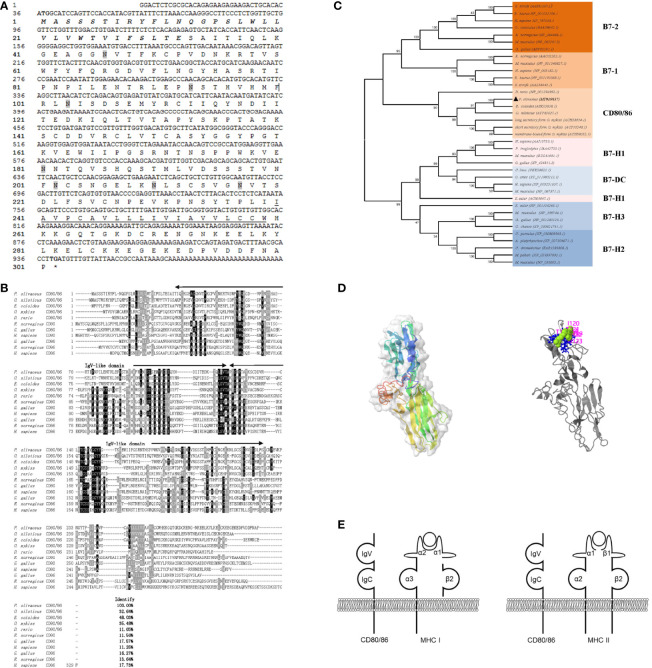
Sequence analysis. **(A)** The sequence of CD80/86 cDNA and deduced amino acid in flounder. The signal peptide and transmembrane domain are in bold italic and underlined, respectively. The N-linked glycosylation sites were shadowed, and the start codon and stop codon are bolded. **(B)** Multiple alignments of CD80/86 protein homologs. The residues that are ≥75% identical among the aligned sequences are in black. The dashes in the amino acid sequences indicate gaps introduced to maximize alignment. **(C)** Phylogenetic trees of B7 superfamily. The unrooted phylogenetic trees are constructed by the neighbor-joining method based on the amino acid alignment (Clustal W) of full-length protein sequences. Numbers in each branch indicate the percentage bootstrap values on 1,000 replicates. The accession numbers were shown behind the species name. **(D)** The prediction of the tertiary structure of CD80/86 and the potential binding site. **(E)** Schematic representation of the distribution pattern of CD80/86 and MHC molecules.

The full-length cDNA consists of a 35-bp 5’ untranslated region (UTR), a 906-bp ORF, and a 54-bp 3’ UTR (GenBank accession No. MT019837). The ORF sequence of *CD80/86* encoded a protein of 301 amino acids with a putative molecular mass of 33.4 kDa with a predicted isoelectric point of 7.43. Seven N-linked glycosylated sites are distributed in the extracellular Ig-like structural domain (**Figure 1A**). The amino acid sequence alignment shows that CD80/86 shares overall 11.56%–48.03% amino acid identity with other teleost’ homologs, and CD80/86 in flounder is more similar to marine fish CD80/86 homologs than that in freshwater fish (**Figure 1B**). The N-J tree was constructed with bootstrap analysis (1,000 replicates) to explore the evolutionary relationship of CD80/86 between teleost and mammals, and the results showed that CD80/86 of flounder clustered into one group in teleost with high bootstrap support and demonstrated exclusivity with other B7 superfamily members, and CD80/86 in fish is closely related to B7-2 (**Figure 1C**).

### The Structure of CD80/86

The structure of CD80/86 was analyzed by SMART (http://smart.emblheidelberg.de/) and Expasy (https://www.expasy.org), as a typical type I transmembrane protein; CD80/86 in flounder is composed of a signal peptide (1–33 aa), two superfamilies’ Ig-like domain (36–130 and 132–224 aa) in the extracellular domain, a transmembrane domain (240–259 aa), and a cytoplasmic region (260–302 aa) (**Figure 1E**). Through homologous modeling using human programmed cell death 1 as templates (PDB Hit: 3FN3, C-score = −3.36, TM-score = 0.34 ± 0.11), a three-dimensional model was constructed (**Figure 1D**). The potential ligand binding site residues were predicted, and the residues 31, 62, 113, 114, 120, 121, 122, and 123 are involved in the binding of CD80/86 to its ligand. These results may indicate that ligand binding site residues mainly distributed in the IgV-like domain participate in ligand binding by folding to form steric structures. Overall, the CD80/86 in flounder was cloned and it shared similar structural features to CD80/86 homologs in other species.

### Tissues and Cellular Distribution of CD80/86

**Figure 2:**
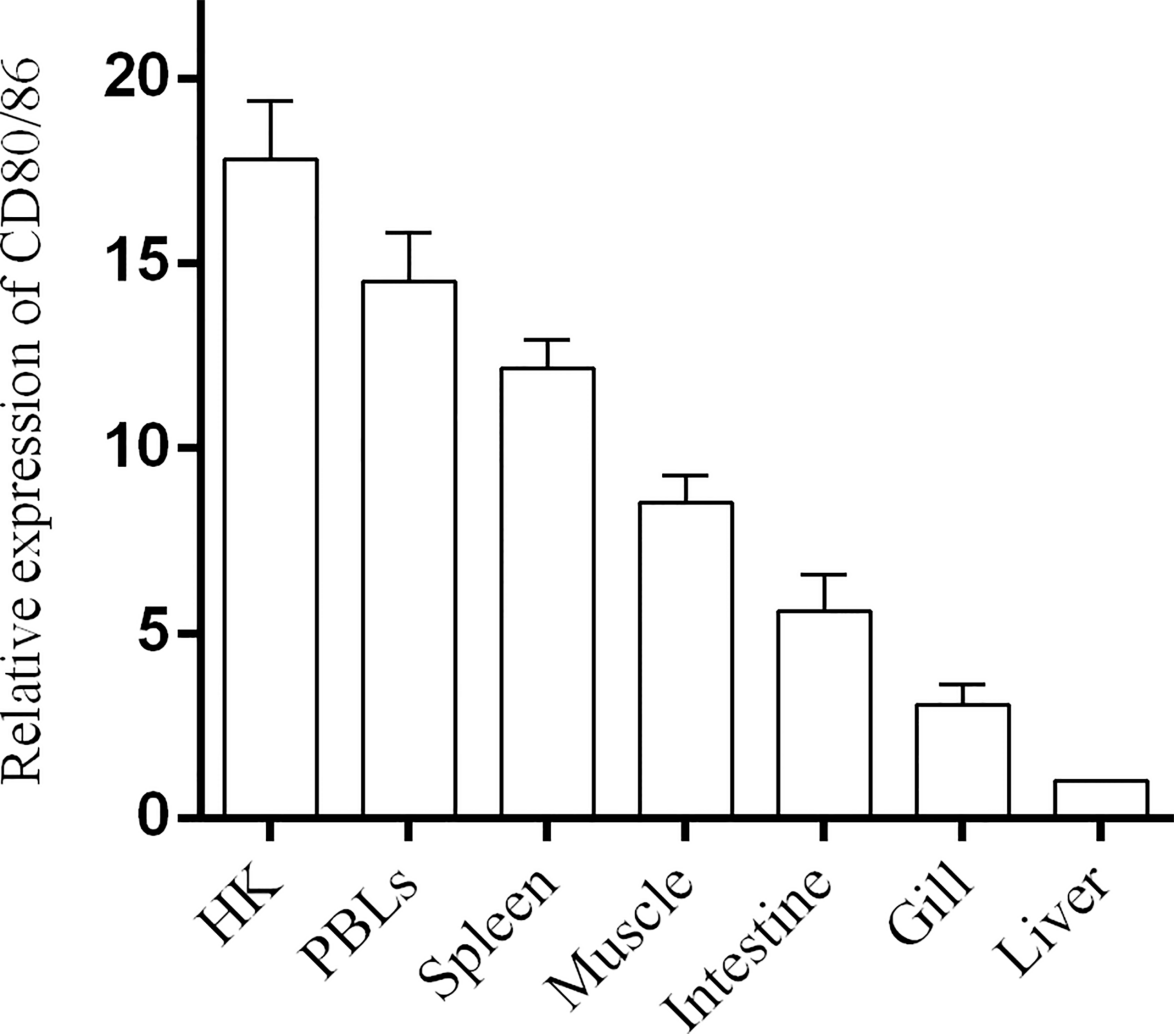
The expression of CD80/86 in flounder. The relative expression level of *CD80/86* in different tissues was normalized to *β-actin* and then compared with the average expression level in the liver in which the expression level was lowest and defined as 1, *n* = 9.

**Figure 3:**
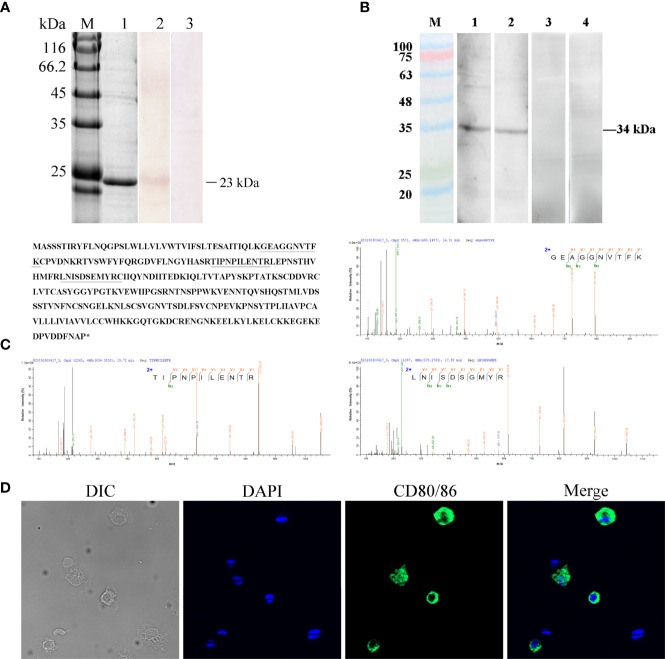
Verification of the specificity of the anti-CD80/86 antibody. **(A)** SDS-PAGE and Western blotting analysis of rabbit anti-CD80/86 antibody with purified recombinant CD80/86 protein. Lane M, protein molecular weight marker; Lane 1, purified recombinant CD80/86 protein; Lane 2, antibodies react to the purified recombinant protein of CD80/86; Lane 3, purified recombinant protein incubated with the immunized serum as a negative control. **(B)** Western blotting analysis of anti-CD80/86 rabbit and mouse polyclonal antibody with the whole-cell protein extracts from PBLs. Lanes 1 and 2, the whole-cell protein extracts from PBLs were incubated with anti-CD80/86 rabbit or mouse polyclonal antibodies, respectively; Lanes 3 and 4, the negative control with unimmunized serum. **(C)** Mass spectrographic analysis of the immunoreactive band at 34 kDa and the matched peptides are underlined and bolded in the amino acid sequence of CD80/86. Asterisk indicates termination codon. **(D)** The localization of CD80/86 on PBLs. Nuclei were stained with DAPI, showing blue fluorescence. Green fluorescence was distributed on the cell membrane. Scale bar = 10 μm.

**Figure 4:**
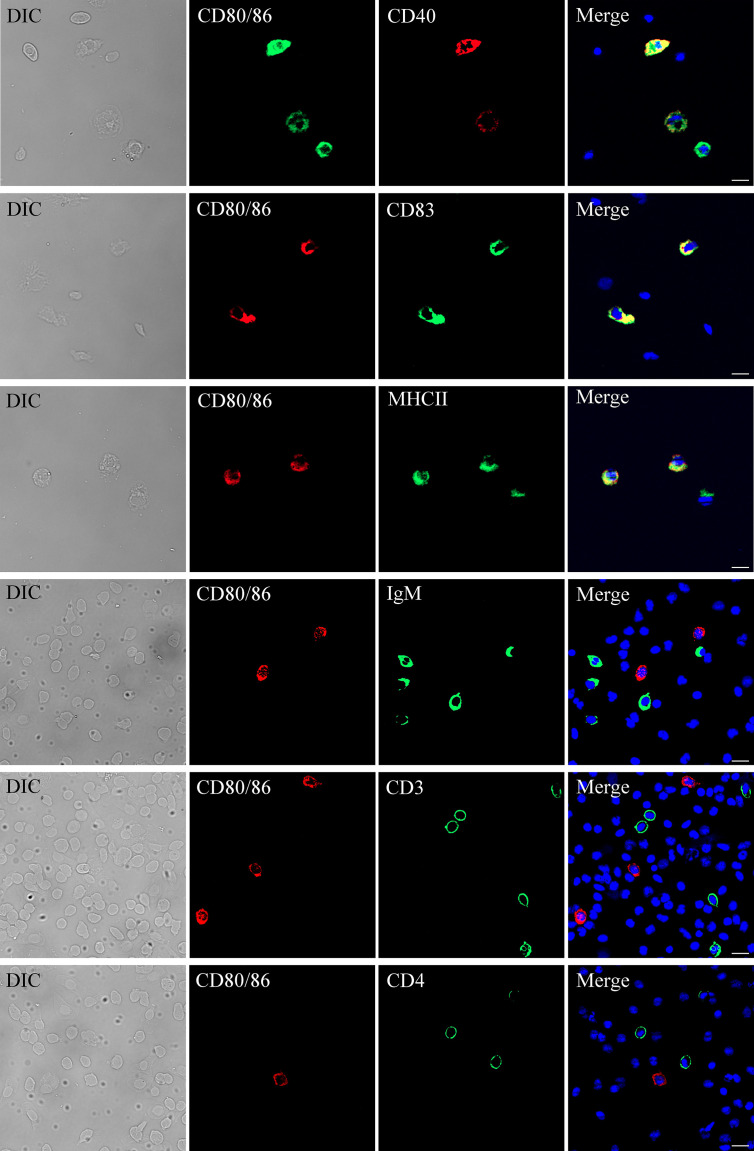
Subcellular localization of CD80/86. The leukocytes were stained with anti-CD80/86 and mouse anti-CD4, rabbit anti-CD3, rabbit anti-CD40, rabbit anti-CD83, rabbit anti-MHCII, or mouse anti-IgM. DAPI staining shows the location of the nucleus. The unimmunized serum was used as negative control (data not shown). The images were obtained using a fluorescence microscope. Scale bar = 10 μm.

The distribution profiles of *CD28* at the transcriptional level were detected by RT-qPCR. Among the seven tissues, namely, the head kidney, spleen, PBLs, muscle, gill, liver, and intestine, the highest expression was detected in the head kidney, followed by the PBLs, spleen, and muscle (**Figure 2**), while the expression of CD80/86 was lowest in the liver. To explore the distribution characteristics of CD80/86 in different cell types, the specific antibodies were produced and the average titers are higher than 1:100,000 (data not shown). The antibodies could react with the purified recombinant protein (~23 kDa) (**Figure 3A**) and a ~34-kDa band with the whole-cell protein extracts of PBLs (**Figure 3B**), which is consistent with the predicted molecular mass of cloned CD80/86. Moreover, the mass spectrometry result showed that 3 peptides were matched to the extracellular sequences (**Figure 3C**). These data suggest that the prepared polyclonal antibodies could recognize the natural CD80/86 in flounder. The isolated leukocytes in peripheral blood are shown in **Supplementary Figure S1**. The distribution of CD80/86 molecules in cell subpopulations of flounder was detected by indirect immunofluorescence staining. Double-immunofluorescence staining results showed that CD80/86 was colocalized with CD83, MHCII, and CD40 on the membrane of PBLs, and not distributed on IgM+, CD3+, or CD4+ lymphocytes (**Figure 4**). Those observations indicate that CD80/86 mainly distribute on the surface of APCs.

### The Expression of DNA Vaccine in Muscle

**Figure 5:**
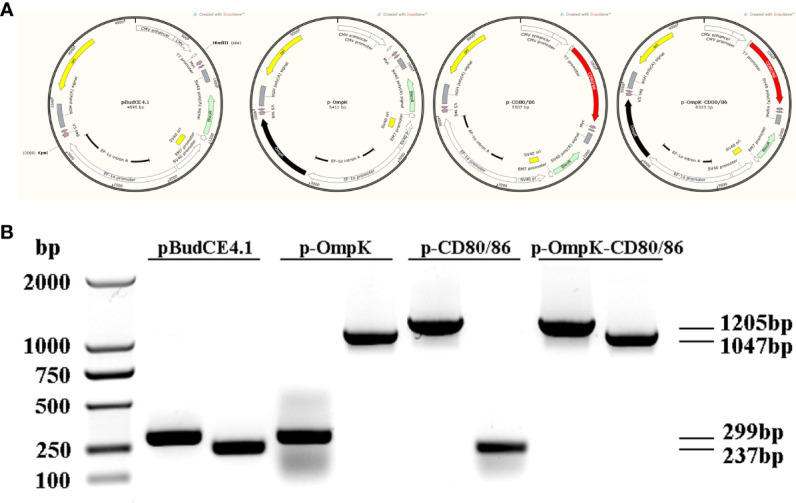
Plasmid construction. **(A)** Structure of recombinant plasmids produced by Snapgene software. The CD80/86 was ligated into the *Hind*III site located downstream of CMV and OmpK was ligated into the *Kpn*I site located downstream of EF-1α, which were represented by a red box and a black box, respectively. **(B)** Agarose gel electrophoresis analysis. The constructed recombinant plasmids were amplified using universal primers of CMV and EF-1α listed in **Table 1**.

**Figure 6:**
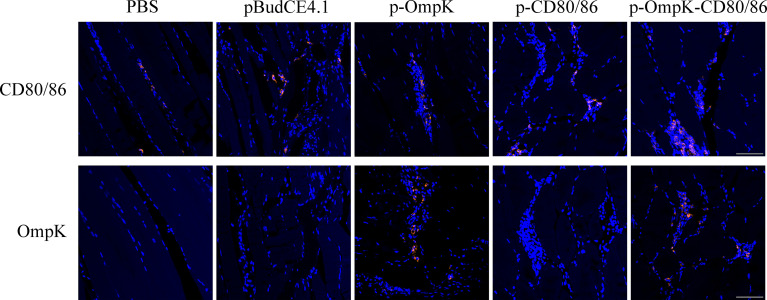
Expression of OmpK and CD80/86 in the muscle of flounder was detected by indirect immunofluorescence on the 7th day after immunization. The muscle from PBS, pBudCE4.1, p-CD80/86, p-OmpK, and p-OmpK-CD80/86 was incubated with rabbit anti-CD80/86 or anti-OmpK antibodies. DAPI staining shows cell nuclei, and the red fluorescence indicated the positive signals of OmpK or CD80/86. Sections from the PBS group were used as the negative control. IIF was analyzed by epifluorescence microscopy at a magnification of 20×. Scale bar = 50 μm.

The bicistronic plasmid, pBudCE4.1, was used to co-express *OmpK* of *V. anguillarum* and *CD80/86* (**Figure 5A**). The recombinant plasmids were amplified with the universal primers (listed in **Table 2**), and specific bands corresponding to the theoretical size bp can be observed in agarose gel (**Figure 5B**). Therefore, recombinant plasmids were successfully constructed and can be used as a vaccine to immunize. The expression of recombinant plasmids *in vivo* was verified by indirect immunofluorescence. After immunization, muscle tissue was sampled from the injection site. Notably, the positive signal of CD80/86 was not only observed in the p-CD80/86 and p-OmpK-CD80/86 groups but also in the pBudCE4.1-, p-OmpK-, and PBS-immunized groups on the 7th day, and more CD80/86 positive signals have been detected in the p-CD80/86 and p-OmpK-CD80/86 groups. After being injected with recombinant plasmids, the specific red fluorescent signal was only detected in the p-OmpK and p-OmpK-CD80/86 groups; no signal was observed in the pBudCE4.1, p-CD80/86, and PBS groups (**Figure 6**). Those observations revealed that the vaccines can be effectively expressed *in vivo*, and the expressed proteins are recognized by prepared specific antibodies.

### Expression of Immune-Related Genes at the Injection Site

**Figure 7:**
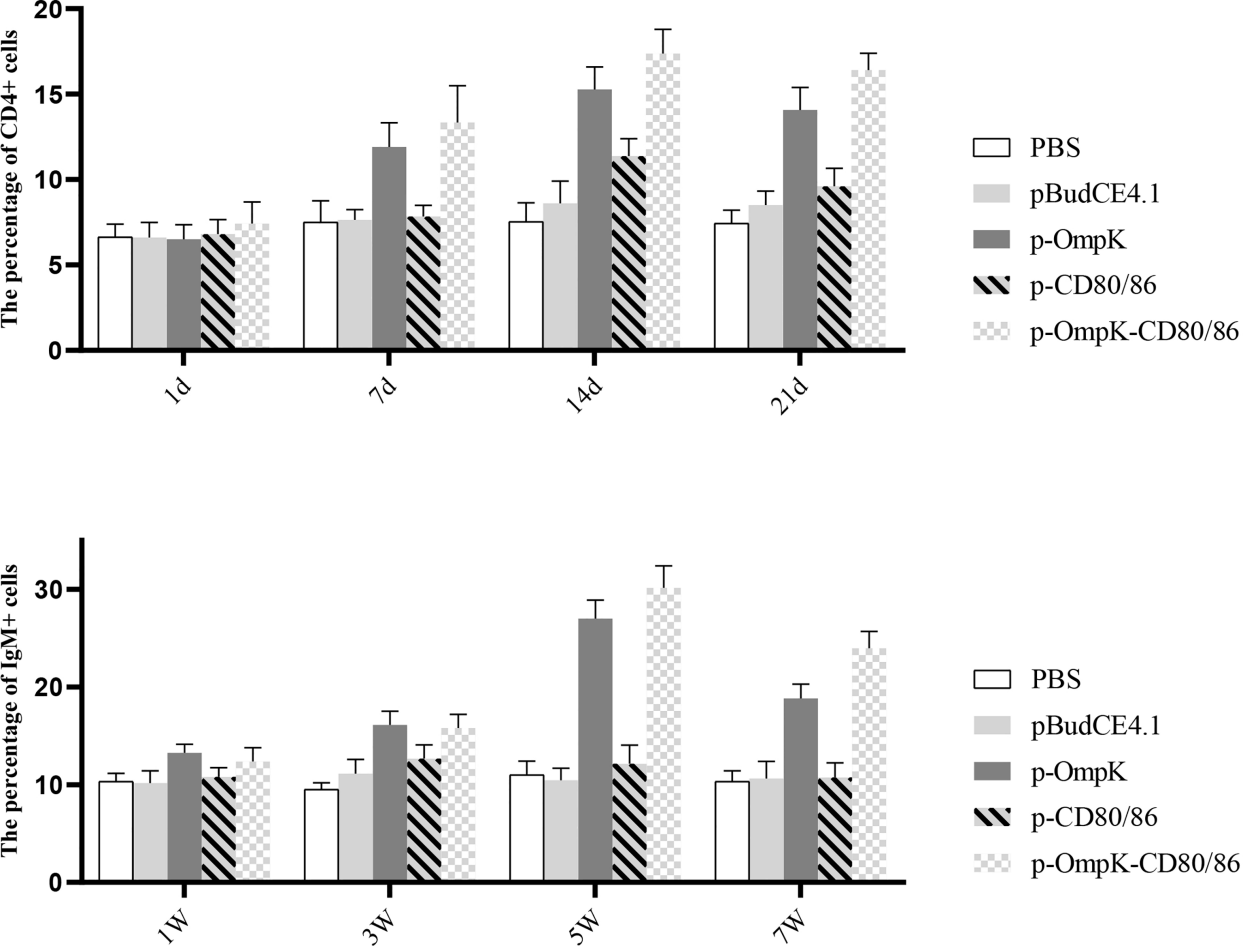
Analysis of the expression profiles of immune-related genes in the muscle around the injection site at the 3rd and 7th day post-immunization detected by qRT-PCR. The expression levels of cytokines IL-2, TNF-α, and IFN-γ; T-cell membrane-bound molecules CD4, CD8, and CD28; and antigen-presenting molecules CD83, MHCI, and MHCII were monitored. The relative expression levels were normalized against *β-actin* and expressed as fold change compared with the PBS-stimulated group at 72 h and 168 h poststimulation. Data were presented as the means ± SD of three fish, and the different letters on the bars indicate the statistical difference (*p* < 0.05) between the experimental and control group at the same time point.

The expression profiles of *CD4-1*, *CD8α*, *CD28*, *TNF-α*, *IFN-γ*, *IL-2*, *CD83*, *MHCIα*, and *MHCIIα* in each experiment group were investigated. The expression of detected genes in the p-OmpK-CD80/86 group was higher than the groups immunized with p-OmpK alone (*p* < 0.05). The expression of *CD4-1*, *CD8α*, *CD28*, and *TNF-α* increased with time and *IL-2* was also increased, induced by p-CD80/86 at 72 h. The higher expression of *CD83*, *MHCIα*, and *MHCIIα*, which are involved in antigen delivery, was detected at 72 h compared to 168 h. As shown in **Figure 7**, the highest *CD28* expression was monitored at 72 h in the pCD80/86 and p-OmpK-CD80/86 groups with no significant difference between the two groups (*p >*0.05) and then decreased at 168 h in the pCD80/86 group.

### Variations of CD4+ and IgM+ Cells in Lymphocytes After Vaccination

**Figure 8:**
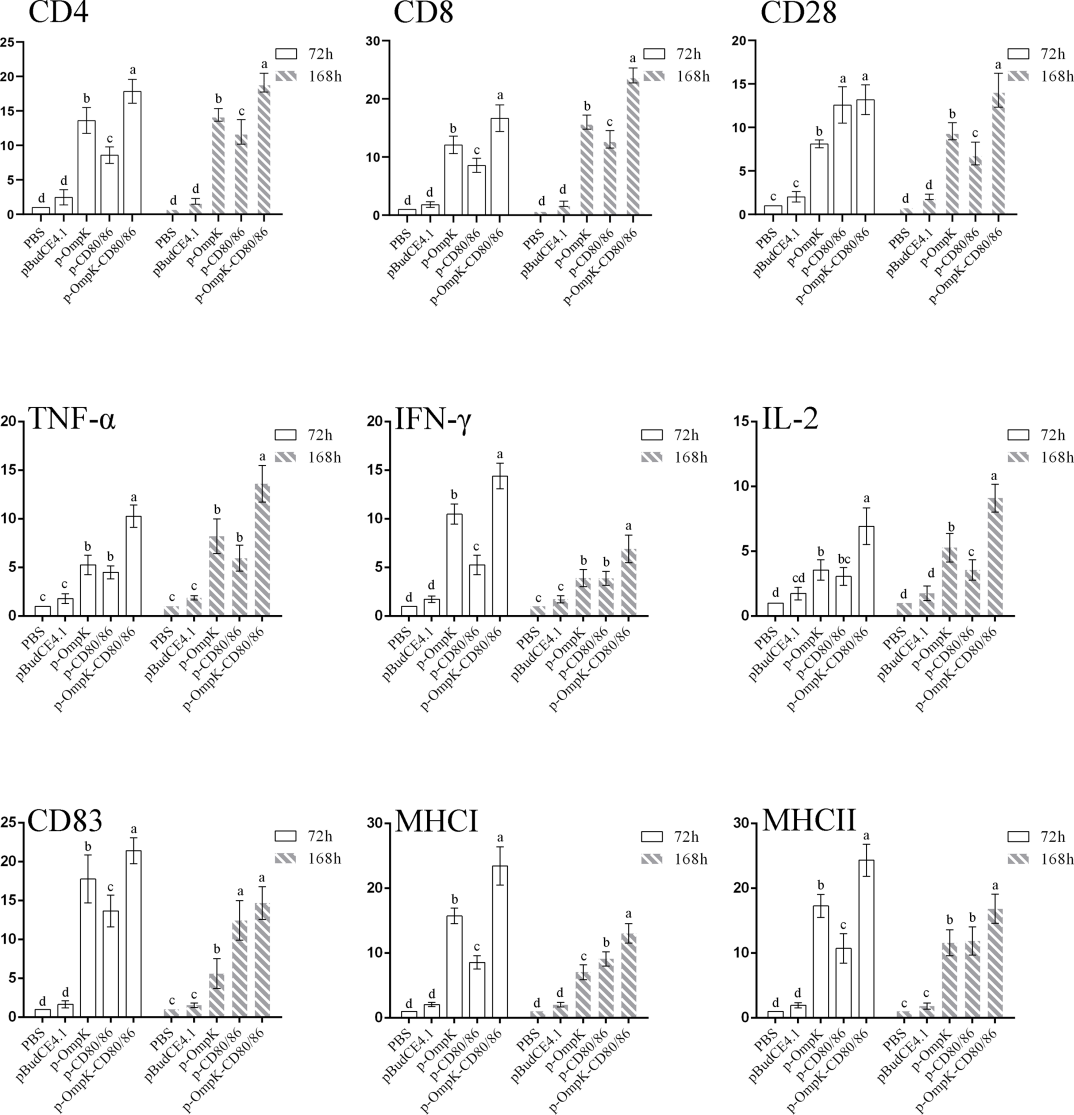
The percentage of CD4+ and IgM+ lymphocytes in peripheral blood of flounder at different time points after immunization. The results are presented as the means ± SD of six fish. Different letters above the bar represent the statistical significance (*p <* 0.05) compared to each other at the same time point.

To further investigate the variations of CD4+ and IgM+ induced by the vaccines, the percentage of T/B lymphocytes was monitored at different time points (**Figure 8** and **Figure S2** in the **Supplementary Material**). Compared to the control group, the percentage of CD4+ cells of peripheral blood lymphocytes increased on the 7th day (*p* < 0.05) and reached the peak on the 14th day with 17.13 ± 1.16% and 18.46 ± 1.06% (*p* < 0.05) in the p-OmpK and p-OmpK-CD80/86 groups. Then, the percentage was slowly decreased on the 21st day, which is still higher than the percentage of CD4+ cells on the 7th day. The percentage of CD4+ cells did not change significantly (*p* > 0.05) among the immunized groups of pBudCE4.1 and p-CD80/86 on the 1st, 7th, and 21st day, while the percentage of p-CD80/86 significantly increased to 11.43 ± 0.70% (*p* < 0.05) on the 14th day compared to the groups immunized with PBS and pBudCE4.1 (7.5 ± 0.86% and 8.6 ± 1.07%). The variations of IgM+ cells showed a similar dynamic profile. In the p-OmpK and p-OmpK-CD80/86 groups, the levels of the IgM+ cell gradually increased, reaching the peak levels at week 5 (27.53 ± 0.98% and 30.36 ± 1.31%, *p*< 0.05), and then decreased slightly. Similarly, the groups immunized with PBS, pBudCE4.1, and p-CD80/86 plasmids did not show significant differences at weeks 1, 2, and 7 (*p >*0.05).

### Immunoprotective Effects Against Challenge With *Vibrio anguillarum*

**Figure 9:**
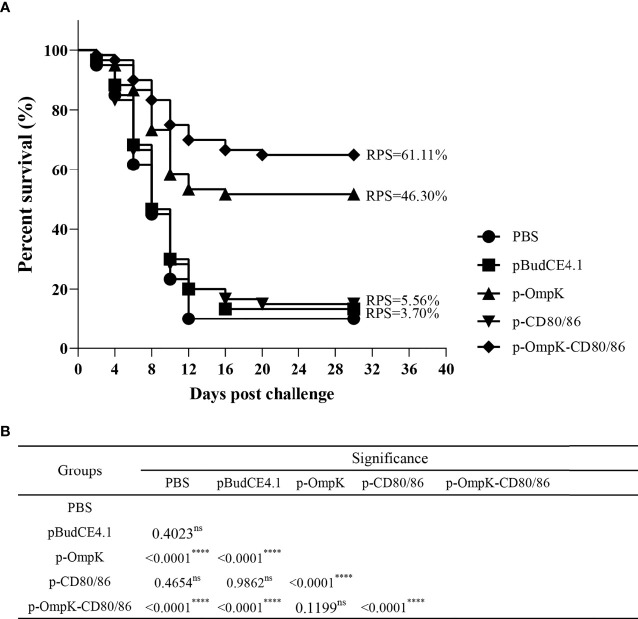
**(A)** The percent survival of immunized flounder after challenge with live *Vibrio anguillarum*. RPS was calculated with a PBS group as the control. **(B)** The log rank method was used to detect any significant difference between the groups, and the “****” indicates a statistically significant difference (*p* < 0.0001), and “ns” indicates no statistically significant difference.

The fish from different immunized groups were challenged with live *V. anguillarum* at week 7. The death of flounder began on day 2, and rapid death occurred on days 5–8. The typical clinical features such as bleeding at the base of the fins and intestinal bleeding appeared on dead fish. After 20 days of challenge, the mortality rate stabilized in each group, and about 85%–90% of the flounder died in the PBS, pBudCE4.1, and p-CD80/86 groups with an RPS of 3.7% and 5.56%, respectively (**Table S1** in the **Supplementary Material**). The vaccine of p-OmpK and p-OmpK-CD80/86 showed effective immune protection against *V. anguillarum* with an RPS of 46.30% and 61.11%, respectively (**Figure 9A**). The mortality rate of each group was analyzed by the log rank method, and the results showed that the mortality rate of the p-OmpK and p-OmpK-CD80/86 groups was significantly different from that of the PBS, pBudCE4.1, and p-CD80/86 groups (**Figure 9B**).

## Discussion

The adaptive immune system originated in jawfish, and many of the key molecules, immune cells, and mechanisms of action involved in adaptive immunity have been preserved during evolution (41, 42). The protein interactions between APCs and T lymphocytes are key events in the induction of T-cell immunity, and several co-stimulatory pathways have been identified in teleost. The interaction of CD40–CD154 plays an essential role in thymus-dependent antibody production (43) and CD58/CD2-mediated co-stimulatory functions involved in the activation of adaptive humoral immunity (44), while the B7-1/B7-2-CD28 co-stimulatory pathway is essential and crucial in regulating T-cell activation by providing the required second signal. The co-stimulatory ligands, CD80 and CD86, expressed on APCs participate in T-cell activation by interacting with CD28 on T cells to prevent anergy (45). However, there are still significant variations in specific molecules between higher and lower species. In this study, only one copy of *CD80/86* was cloned in flounder and the phylogenetic analysis showed that CD80/86 in bony fish may have a close relationship to CD86 than CD80 among mammals and birds.

Although there was less than 20% sequence identity in amino acid sequence comparisons between bony fish and mammals, the conserved structural features were shared. The CD80/86 protein in flounder comprises two extracellular Ig-like domains (36–130 and 132–224 aa). The Programmed cell death 1 ligand (PD-L1), also known as B7-H1, belongs to the B7 superfamily, which also contains conserved extracellular IgV-like and IgC-like domains (10) and can be used as the template to construct the 3D structure of CD80/86 with the optimal C-score. The 3D structure of CD80/86 in flounder contains two Ig-like domains, and the binding site can be found in the IgV-like domains, which indicated that IgV-like domains may be involved in ligand recognition; IgC-like domains may be used to maintain the CD80/86 structure. Corresponding to this result, N-glycosylation sites were found in the two extracellular Ig-like domains and mainly in IgC-like domains, and glycosylation modifications play an important role in the maintenance of immunoglobulin structure and activity stability (46). Consistent with this observation in other bony fish, the results showed that similar extracellular structural features and N-glycosylation modifications with different amounts were shared (20, 22, 47).

Tissue distribution analysis shows that CD80/86 is mainly expressed in immune-related tissues, especially in the head kidney, PBLs, and spleen. Despite the fact that CD80/86 transcript levels differed greatly among tissues, similar expression characteristics were reported in rainbow trout, in which the highest expression of rtCD80/86A was detected in the thymus, followed by the spleen and blood (48). Interestingly, higher levels of CD80/86 expression were also found in muscle tissue of rainbow trout (rtCD80/86A, rtCD80/86B, and rtCD80/86ss), yellow catfish, and Nile tilapia. It is widely accepted that muscle is an important unit for maintaining body movement, and more studies have reported on the immune functions of muscle, which are referred to as non-professional APCs to maintain muscle-directed immune responses (49). Under inflammatory conditions, TLRs are activated in muscle, which triggers the production of cytokines and chemokines, all of which can orchestrate the recruitment of leukocytes for innate and adaptive immune responses (50, 51).

Corresponding to the structural analysis of CD80/86, the CD80/86 in flounder was detected on the membrane surface of PBLs with specific antibodies and the double-immunofluorescence staining revealed that CD80/86 colocalized with CD83, MHCII, and CD40 molecules on leukocytes and no CD28-positive signal was detected on IgM+, CD3+, or CD4+ cells. In teleost fish, the head kidney of fish undertakes the hematopoietic function, and it is the main immune organ responsible for phagocytosis, antigen processing, and formation of IgM and immune memory through melanomacrophagic centers (52, 53). We have proved that CD80/86 was colocalized with MHCII, CD40, and CD83 on leukocytes. Therefore, the higher expression of CD80 in HK than in PBLs is associated with its distribution in APCs. In the zebrafish model, CD80/86 colocalized with mIgM on B cells and with MHCII on DCs, which are the most important APCs (22, 54). As reported in humans and mice, CD80 was expressed in T cells, B cells, DCs, and monocytes after induction, whereas CD86 was constitutively expressed in B cells, DCs, and monocytes, as well as in induced T cells (10). Based on similar distribution characterization and structural properties, CD80/86 is conserved in bony fish and has a closer relationship to CD86 counterparts.

*V. anguillarum* is a serious pathogenic bacteria in aquaculture that caused massive mortality of marine fish, bivalves, and crustaceans (55, 56). More evidence proved that the outer membrane proteins of pathogenic bacteria are highly immunogenic and provide immune protection against pathogenic bacteria in fish as a vaccine (57–59). In our previous research, multiple immunogenic proteins have been identified in *V. anguillarum* including VirA, CheR, FlaC, OmpK, OmpR, Hsp33, VAA, Groel, OmpU, PteF, and SpK (60, 61); the OmpK and OmpR as subunit vaccines induced the secretion of specific antibodies and immunoprotection with an RPS of 62.16% and 64.86%, respectively, against *V. anguillarum*. To enhance the protective effect of OmpK as a DNA vaccine candidate, adjuvants were co-administered with the vaccine. The previous study has shown that two chemokines, CCL4 and CCL19, were inserted into the OmpK-based DNA vaccine (39) and provided RPS of 74.1% and 63.3%, respectively. Furthermore, the leukocytes were recruited to the injection site after immunization. The mechanisms of action of adjuvants include the slow release of antigen, secretion of cytokines and chemokines, recruitment of APCs, and increased inflammatory response at the injection site (62). T-cell activation is the key to inducing cellular immunity and the B7-CD28 pathway is the key to T-cell activation. Unlike chemokines, which recruit leukocytes to the immunological site, the co-stimulatory molecules may promote the recognition of antigens by the acquired immune system through affecting the interactions between APCs and T cells to produce specific antibodies and cellular responses. After being immunized with p-OmpK-CD80/86, the expression of *CD4* and *CD8* in muscle and the induced percentage of IgM+ and CD4+ lymphocytes in peripheral blood are higher than induced by p-OmpK alone, while this phenomenon is not caused by CD80/86. These data suggest that p-OmpK-CD80/86 can induce a rapid and strong protective immune response against *V. anguillarum*. In mice, delayed-type hypersensitivity and cytotoxic T lymphocyte (CTL) activity were significantly enhanced when the B7-2-expressing plasmid was co-inoculated with plasmids encoding HIV-1 env and rev, but were not affected when the B7-1-expressing plasmid was used with the vaccine (63). Similarly, the co-stimulatory molecule OX40L enhances HBV DNA vaccine-induced humoral immune response and also enhances CTL-mediated specific cellular immune responses (64). Interestingly, we observed that plasmids p-OmpK-CD80/86 and p-CD80/86 were able to significantly upregulate the expression of CD28 and IL-2 at 72 h after immunization. As demonstrated in Nile tilapia, CD28 is able to bind to CD80/86 (21). Therefore, we speculated that the expression of CD80/86 activated the CD28 signaling pathway, which caused activation of T cells and consequently increased IL-2 expression. However, there are few applications of B7 molecules as adjuvants, and further work is needed to explore the possibility of B7 as other types of adjuvants and the specific mechanism of action.

In conclusion, our data demonstrate that CD80/86 in flounder is conserved to its homologs in fish and mammals and clarify its distribution characteristics in fish lymphocyte subsets. In addition, the efficiency of CD80/86 as an adjuvant in DNA vaccine was evaluated in fish. Furthermore, this study first reported the possibility of co-stimulation as an adjuvant for fish vaccines, providing basic data for the development and application of more types of vaccine adjuvants.

## Data Availability Statement

The datasets presented in this study can be found in online repositories. The names of the repository/repositories and accession number(s) can be found at: https://www.ncbi.nlm.nih.gov/nuccore/MT019837.1/.

## Ethics Statement

All experiments were strictly conducted according to the procedures described in the Guide for the Use of Experimental Animals of the Ocean University of China, in agreement with the International Guiding Principles for Biomedical Research Involving Animals (EU84 2010/63). All efforts were dedicated to minimizing suffering.

## Author Contributions

Designed the experiments: WL, JX, and WZ. Performed the experiments: WL. Analyzed the data: WL, HC, and JX. Provided reagents/materials/analysis tools: XT and XS. Wrote the manuscript: WL, JX, and WZ. All authors contributed to the article and approved the submitted version.

## Funding

This work was supported by the National Key Research and Development Program of China (2018YFD0900503), the National Natural Science Foundation of China (32173005, 31730101, 31672684) and the Shandong Provincial Natural Science Foundation (ZR2020KC025).

## Conflict of Interest

The authors declare that the research was conducted in the absence of any commercial or financial relationships that could be construed as a potential conflict of interest.

## Publisher’s Note

All claims expressed in this article are solely those of the authors and do not necessarily represent those of their affiliated organizations, or those of the publisher, the editors and the reviewers. Any product that may be evaluated in this article, or claim that may be made by its manufacturer, is not guaranteed or endorsed by the publisher.
