# Supplementary material for: Characterization of Co-Stimulatory Ligand CD80/86 and Its Effect as a Molecular Adjuvant on DNA Vaccine Against Vibrio anguillarum in Flounder (Paralichthys olivaceus)
Source: Front Immunol. 2022 May 10;13:881753. doi: 10.3389/fimmu.2022.881753 (PMC9127221; doi:10.3389/fimmu.2022.881753)
Supplement: Supplementary file 1 [file DataSheet_1.docx]

**Characterization of co-stimulatory ligand CD80/86 and its effect as a molecular adjuvant on DNA vaccine against *Vibrio anguillarum* in flounder (*Paralichthys olivaceus*)**

Wenjing Liu^1^, Jing Xing^1,2*^, Xiaoqian Tang^1,2^, Xiuzhen Sheng^1^, Heng Chi^1^, Wenbin Zhan^1,2^

^1^ Laboratory of Pathology and Immunology of Aquatic Animals, KLMME, Ocean University of China, Qingdao 266003, China

^2^ Laboratory for Marine Fisheries Science and Food Production Processes, Qingdao National Laboratory for Marine Science and Technology, No. 1 Wenhai Road, Aoshanwei Town, Qingdao, China

*Corresponding authors: [xingjing@ouc.edu.cn](mailto:xingjing@ouc.edu.cn)

Address: 5 Yushan Road, Qingdao, Shandong Province, China

Tel: +86-532-82032284; fax: +86-532-82032284

| Table1 The cumulative number of deaths on different dates | | | | | | | | | | | |
| --- | --- | --- | --- | --- | --- | --- | --- | --- | --- | --- | --- |
|  | Days | | | | | | | | | | |
| Groups | 0 | 2 | 4 | 6 | 8 | 10 | 12 | 16 | 20 | 24 | 30 |
| PBS | 0 | 3 | 9 | 23 | 33 | 46 | 54 | 0 | 0 | 0 | 0 |
| pBudCE4.1 | 0 | 2 | 7 | 19 | 32 | 42 | 48 | 52 | 0 | 0 | 0 |
| p-OmpK | 0 | 1 | 3 | 8 | 16 | 25 | 28 | 29 | 0 | 0 | 0 |
| p-CD80/86 | 0 | 2 | 10 | 20 | 32 | 43 | 48 | 50 | 51 | 0 | 0 |
| p-OmpK-CD80/86 | 0 | 1 | 2 | 6 | 10 | 15 | 18 | 20 | 21 | 0 | 0 |


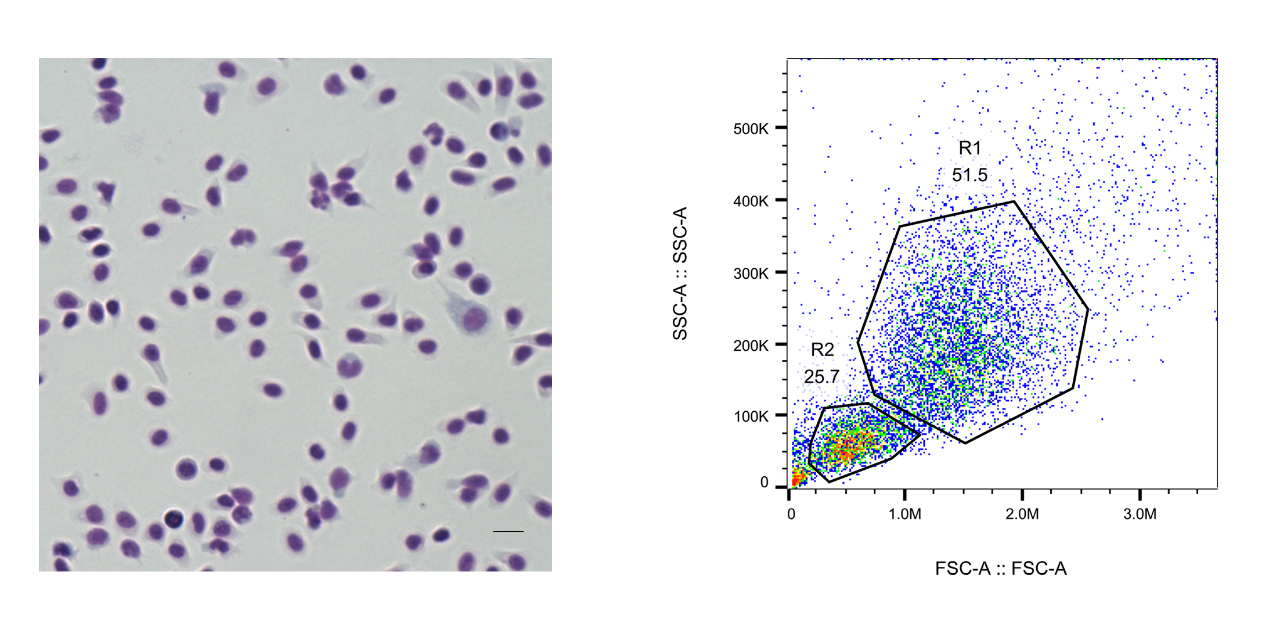


**Supplemental Figure S1**. Assessment of the leukocytes in peripheral blood. Wright–Giemsa staining shows the morphological characteristics of leukocytes and FCM analyzed using a FSC–SSC dot plot, scale bars=10 μm.


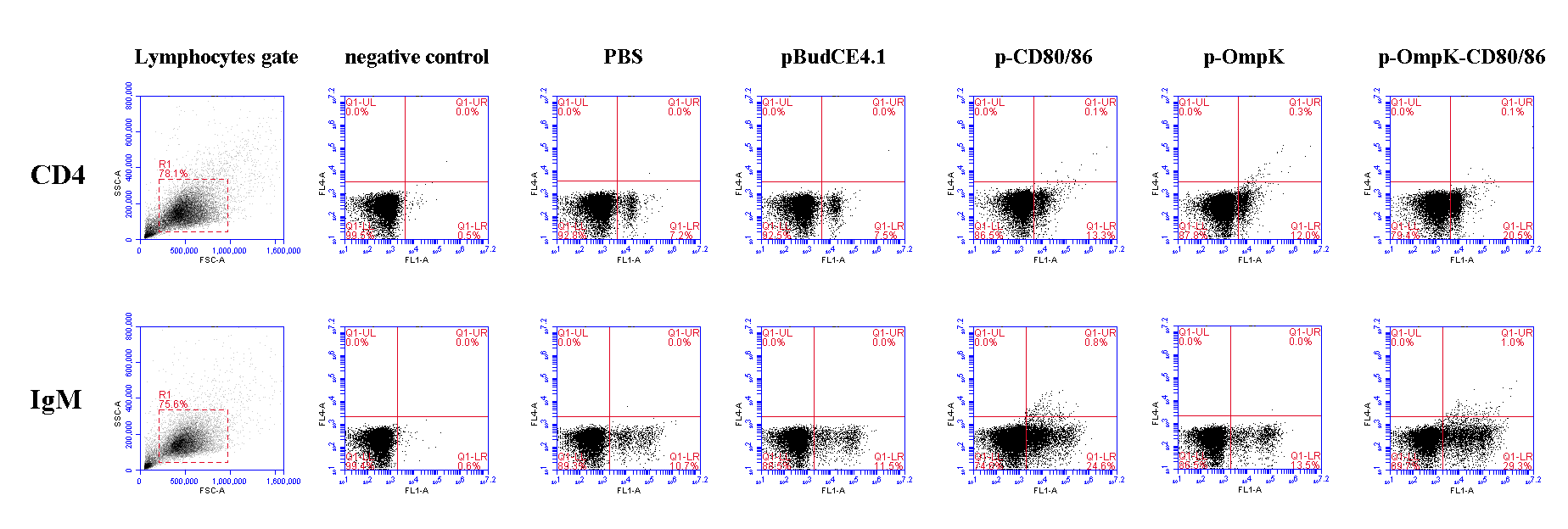
 **Supplemental** **Figure S2**. The percentage of CD4+ and IgM+ lymphocytes in peripheral blood of flounder after immunization. **Lymphocytes gate**: Side-scatter (SSC)/forward-scatter (FSC) dot plots in peripheral blood lymphocytes. **CD4**: lymphocytes in peripheral blood were incubated with mouse anti-CD4 antibodies as primary antibody at 7^th^ day after immunization with different stimulation (PBS, pBudCE4.1, p-CD80/86, p-OmpK and p-OmpK-CD80/86). **IgM**: lymphocytes in peripheral blood were incubated with mouse anti-IgM antibodies as primary antibody at 5 w after immunization with different stimulation (PBS, pBudCE4.1, p-CD80/86, p-OmpK and p-OmpK-CD80/86). **Negative control**: the unimmunized mouse serum was used as primary antibody.
